# Validity and reliability of Polar M400 GPS watches for measuring distances covered by team sports players

**DOI:** 10.1016/j.heliyon.2023.e20920

**Published:** 2023-10-14

**Authors:** Piotr Makar, Adam Kawczyński, Rui Miguel Silva, Mehmet Yildiz, Ana Filipa Silva, Zeki Akyildiz

**Affiliations:** a Gdańsk University of Physical Education and Sport, Poland; b Escola Superior Desporto e Lazer, Instituto Politécnico de Viana do Castelo, Rua Escola Industrial e Comercial de Nun’Álvares, 4900-347 Viana do Castelo, Portugal; c Research Center in Sports Performance, Recreation, Innovation and Technology (SPRINT), 4960-320 Melgaço, Portugal; d Afyon Kocatepe University Sports Science Faculty, Turkey; e The Research Centre in Sports Sciences, Health Sciences and Human Development (CIDESD), 5001-801 Vila Real, Portugal; f Faculty of Sport Sciences, Gazi University, 06560 Ankara, Turkey

**Keywords:** Sport watches, Assessment, Reliability

## Abstract

Monitoring locomotor demands in team sports becomes popular in professional and recreational daily activities. Precise measurements are main of importance in training routine. The aim of this study is three-fold: (i) analyze the validity of Polar M400 Global Positioning System (GPS) watches for measuring distances covered in team sports simulation cycle; (ii) testing inter-unit reliability of two devices attached during testing; and (iii) testing inter-session reliability for the same simulation cycle. **Methods**: Twenty-one team athletes (age: 24.5 ± 5.2 years; body mass: 71.8 ± 5.7 kg; height: 176.5 ± 4.3 cm) were tested in the team sport simulation cycle (TSSC). Two Polar M400 sport watches were used by each player on their wrists at the same time. The data obtained from Polar M400 were compared to the reference fixed distance of the TSSC to determine the watch validity. Inter-session reliability was also tested using the two watches in two different sessions. **Results:** No significant differences between the reference value and the watches (F = 1.086; p = 0.368; $\eta_{p}^{2}$ ≤0.042) were found. The %CV (0.03–0.05%) and SEM (0.05–0.09) values found for all considered groups confirmed good levels of reliability of the Polar M400 to measure total distance. **Conclusions:** The Polar M400 is a valid and reliable watch for measuring the distances covered by team sport athletes. Both coaches and athletes can monitor the distances covered with accuracy and precision, through the use of the Polar M400 sport watch.

## 1 Introduction

Monitoring locomotor demands in team sports becomes popular in daily coaching activities [1]. One of the main reasons for monitoring locomotor demands is to quantify and qualify the implication of the training programme on the physical responses of the players [2]. Since most of the activities are dependent on drill-based tasks, there is no other objective way to quantify the physical impact [3]. This quantification allows coaches to understand the variability of physical impact between players and individualize the training process [4]. Additionally, daily monitoring of locomotor demands also allows identifying the progression in training load imposed week by week aiming to understand the impact of training across the time [5]. Measuring the total distance traveled by athletes in training and competitions is of great importance for understanding the workload [6]. Different devices are applied in soccer and other team games as an important part of training routine and planning [7,8].This quantification can enhance the load management and avoid exposure to under or over-stimulus to the players [9].

The evolution of microelectromechanical systems (MEMS) and in particular Global Positioning Systems (10.13039/100011109GPS) can support the justification for the massive extension of locomotor demands monitoring in team sports [10]. The development of easy-to-use GPS systems allows for applying tracking systems anywhere in a low-cost and nonintrusive fashion [9]. The GPS becomes popular because does not require fixed installation (such as optical systems or Local Positioning Systems) and is particularly interesting since can monitor any outdoor activities with acceptable accuracy and precision [11].

However, using GPS devices requires trustable accuracy and precision levels aiming to avoid bias about the data collection. For example, a low level of precision may provide a bias in the interpretation of human performance, namely adaptations [12]. In fact, looking for human adaptations, it is important to be aware of the smallest meaningful changes [13]. If the system fails to be reliable and precise, it is not possible to know if the variability reported is caused for the systems or for human performance. On the other hand, guaranteeing the accuracy and validity of the system is also a determinant of performing accurate benchmarks and having the exact idea of the demands occurring [14].

Team sports players normally use GPS sensors integrated with inertial measurement units positioned on the chest [15] or in between scapulae [16]. The systems are fixed to vests and a fixed bag. It is well-known that GPS devices for team sports (>10Hz) are accurate and precise for registering total distances and distances covered to different speed thresholds [11]. Despite the increase in the error in high-intensity demands, presents acceptable values of accuracy and precision [11]. Different systems and brands (e.g., Polar Team pro, Johan Sports) were tested and identified as valid and reliable [[15], [16], [17]].

However, using GPS watches is not so common in team sports. The positioning of the sensor is different and the availability of information in team sports is not so high. In this case, the Polar M400 GPS is a watch with 2.95x3.74 × 4.53 inches, with a GPS integrated which claims to accurately detect altitude, ascent, descent, distance, and speed/pace, namely providing accuracy of ±2 % in distance and ±2 km/h in speed. Although research about validity and reliability of the heart rate (HR) sensor of Polar M400 GPS [18,19], there is no research reporting the validity and reliability of the GPS measures extracted from the device.

Since sports scientists and coaches are using devices such as Polar M400 GPS for monitoring locomotor demands of athletes and to make decisions regarding training organization and stimulus provided, it is vital to certify the validity and reliability of the device. It must be underlined that experiment aimed on devices validity and reliability should be based on well-designed test used in sport, especially in team sport. Test should be physiologically connected with loads during training and competition [[20], [21], [22]] [[20], [21], [22]] [[20], [21], [22]].

This will help to identify if the values are accurate and precise and allow coaches to focus on the human variability or, at least, mitigate the bias of data collection. Based on that, this study aimed to: (i) analyze the validity of Polar M400 Global Positioning System (GPS) watches for measuring distances covered in team sports simulation cycle; (ii) test the inter-unit reliability of two devices attached during testing; and (iii) test the inter-session reliability for the same simulation cycle.

## 2 Methods

### 2.1 Study design

**Fig. 1:**
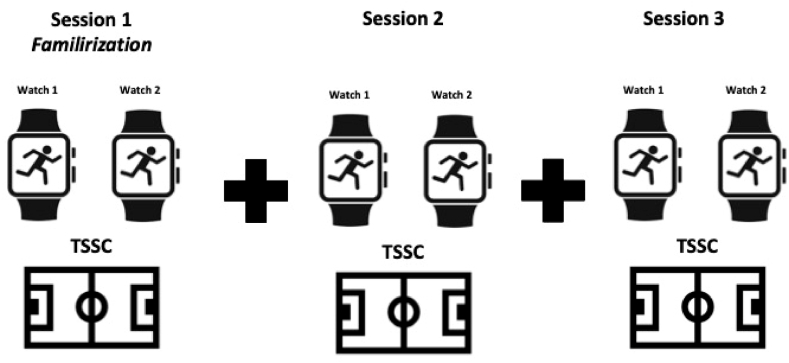
Protocol design.

This study followed an observational cross-sectional design. This study was designed to examine the validity and reliability of the data obtained from GPS watches worn by athletes in Team Sport Simulation Cycle (TSSC). The actual running distances in this study were used to examine the validity of the research. The actual distance of 1200 m of TSSC and the distances obtained from the watches were compared for validity. Regarding the reliability, two different forms of reliability were examined. The first reliability was the intra-unit reliability of the watches and the second reliability was the inter-session reliability of the watches. To examine intra-unit reliabilities, both watches were worn simultaneously by the same athlete during the TSSC protocol. The data obtained from the watches were compared for intra-unit reliability. To examine inter-session reliability values, measurements were repeated on different days in the TSSC protocol. In this way, both the intra-unit reliability between the watches and the measurement reliability of the watches on different days under the same conditions were examined (See Fig. 1).

### 2.2 Setting and context

A 24-h rest was done before the tests. Data were conducted on Monday, Tuesday, and Wednesday of the week; the tests were done in the afternoon; data collection period was between 17:00–19:00 h of the day; temperature: 28 °C averages; relative humidity: averaged 55 %. All measurements were made on natural turf floor in the stadium. Research measurements were obtained at the end of the season of the athletes included in the study. The people who performed the research measurement were the same in all sessions and were experts with a PhD degree in sport science.

## 3 Participants

The sample size was calculated a priori in G*power (version 3.1.9.2; Kiel University, Kiel, Germany). Effect size f = 0.8; α err prob = 0,05; Power (1-β err prob) = 0,7; Number of groups = 4; Actual power = 0,77 the recommended total sample was 20. The inclusion criteria were: (i) no missing data (being part of all assessments); (ii) not be injured or sick in the two weeks preceding and during the observation period; and (iii) not using any medication or similar substance. Participants who did not meet these characteristics were excluded from the study. Twenty-one team athletes (age: 24.5 ± 5.2 years; body mass: 71.8 ± 5.7 kg; height: 176.5 ± 4.3 cm) voluntarily participated in this study. The participants consisted of soccer team athletes who train regularly. All participants were players of the same soccer team. The athletes were a well-trained group. The average years of sporting experience of the athletes was 5 years. They trained at least 5 days a week and participated in official competitions at the weekend. The athletes included in the study played 1 competition per week.

TSSC was explained to the subjects 24 h before the test day when the data was recorded, and they were applied to familiarize them with the TSSC protocol.

### 3.1 Experimental approach

This study is to investigate the validity, intra-unit reliability and inter-session reliability of Polar M400 GPS watches for total distance covered measurements. The measurements were divided into four separate groups to see the differences between the different sessions and the two separate GPS watches. First group; Session-1- GPS Watch - 1, second group; Session-1-GPS Watch - 2, third group; Session-2- GPS Watch - 1, fourth group; Session-2-GPS Watch - 2 has been divided into four separate groups.

During the tests, the athletes wore two Polar GPS watches on their wrists at the same time. The tests of this research took a total of three days. Athletes came for the familiarization phase on the first day. On the second and third days, all athletes were given team sports stimulation in the team sports simulation cycle (TSSC) protocol.

### 3.2 Team Sport Simulation Cycle

**Fig. 2:**
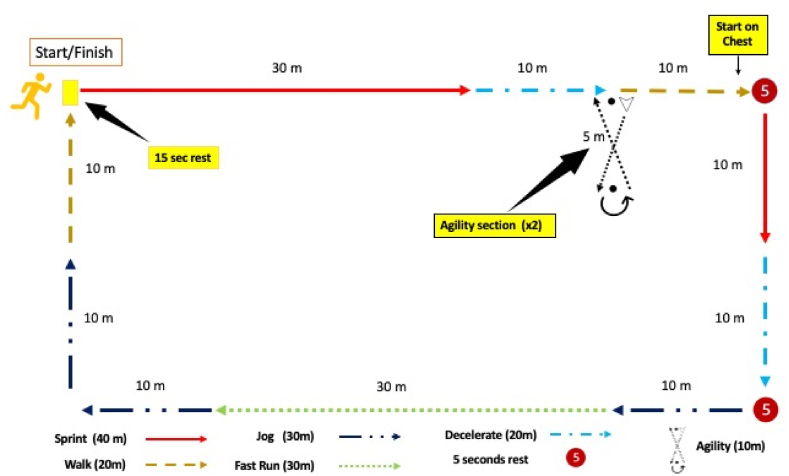
The Team Sport Simulation Cycle is used to determine the validity and reliability of Polar M400 GPS watches.

The TSSC protocol applied to the athletes included acceleration, deceleration, sprinting, walking, jogging, and change of direction [23,24]. The distances of the TSSC protocol were previously measured with a tape measure. The previously measured distance was accepted as the benchmark. The TSSC protocol consists of 8 turns in total. When the entire TSSC is finished, 1200 m are completed/reference value) [23,24]. The movements within the TSSC protocol simulate team sports. The TSSC is shown in detail in Fig. 2. The Team Sport Simulation Circuit (TSSC) was meticulously designed and meticulously executed to replicate the dynamic and multifaceted movements commonly observed in team sports. It encompassed a comprehensive range of athletic actions, including swift accelerations, rapid decelerations, sudden stops, steady walking, and the vigorous sprinting characteristic of team sport athletes. During the installation phase of the TSSC, precise measurements were taken using a tape measure, establishing a benchmark distance of 150 m. This benchmark distance served as the gold standard for calculating the total distance covered by the GPS units worn by the participants during the circuit. The TSSC itself featured a total course length of 1200 m. To complete the TSSC, each participant was tasked with completing eight laps, with each lap measuring precisely 150 m. The primary aim of this protocol was to provide athletes with a realistic platform to simulate the diverse movements and scenarios frequently encountered in team sports.

The TSSC protocol was executed on a regulation soccer field during the afternoon, and the tests were conducted under 3G soccer field conditions. The meteorological conditions on the test day were notable, with an average temperature of 28 °C and an average relative humidity of 55 %. This meteorological data was sourced from regional weather reports, ensuring accuracy in environmental conditions. In preparation for the TSSC protocol, participants were given detailed instructions to maintain optimal physical condition for the test. They were advised to ensure sufficient fluid intake and adhere to a well-balanced diet in the 24 h leading up to the test. Moreover, participants were cautioned against engaging in strenuous physical activities, as well as abstaining from alcohol and caffeine consumption during this period. A key element in the pre-circuit routine was a standard warm-up session, which was thoughtfully designed to mitigate the risk of injuries. Under the vigilant supervision of a qualified researcher, players embarked on a gradual warm-up, including submaximal running, to gradually increase their body temperature and activate key muscle groups. This preliminary phase, which lasted approximately 5 min, played a pivotal role in preparing the athletes for the demands of the circuit. Notably, soccer players adhered to the FIFA 11+ warm-up protocol, a comprehensive regimen that effectively raised body temperature, engaged muscle groups, mobilized joints, and maximized the athlete's physical potential. The FIFA 11+ component of the warm-up routine was allocated about 10 min for completion. All warm-up sessions were conducted on an individual basis, ensuring that each player received personal attention and tailored guidance from the supervising researcher. This approach also facilitated a smooth transition for players as they moved from the warm-up phase to the TSSC protocol, ultimately contributing to the safety and effectiveness of the entire testing process.

## 4 Polar M400 GPS

Athletes wore two Polar M400 GPS watches during the TSSC protocol. Polar M400 GPS (Polar GPS, Polar Electro, Kempele, Finland) watches weigh 56.6 g and are 11.5 mm thick. It can provide Bluetooth Smart connection with mobile phone and sensors. GPS sampling rate is 1 Hz per second. Polar Flow web service compatibility and Polar Flow mobile app compatibility with PC Windows XP, Windows 7, Windows 8 or later. Data from M400 GPS watches used in TSSC protocol were transferred to cloud storage area via Bluetooth. Data was converted from the cloud system in Microsoft excel format.

### 4.1 Statistical procedures

The normality of the data obtained from the GPS watches was tested with the Shapiro-Wilk test. The Shapiro-Wilk test was accepted as the most appropriate test of normality for the sample size [25]. The measurements were divided into four separate groups to see the differences between the different sessions and the two separate GPS watches. First group; Session-1- GPS Watch - 1, second group; Session-1-GPS Watch - 2, third group; Session-2- GPS Watch - 1, fourth group; Session-2-GPS Watch - 2 has been divided into four separate groups. One-way ANOVA test was used to see the differences between the groups. Bonferroni homoscedasticity were used.

One Way ANOVA with Bonferroni post hoc test was used to compare differences between the different sessions and the two separate GPS watches. Percentage coefficients of variation (%CV) were calculated using the formula standard error of mean/mean*100 [26,27]. CV% and SWC tests were used to measure the intra-GPS watch's reliability of the data. The validity of the differences in total distance covered of the GPS watches was analyzed via the Bland-Altman plot. The CV was classified as good (<5 %), moderate (5%–10 %), or poor (>10 %) [11,28,29]. Eta squared values were also reported for the effect sizes [30]. All data processing and analyses were conducted with the R programming language. p values lower than 0.05 were considered significant. All data is available as open access (Access link: https://osf.io/f2u78/; https://doi.org/10.17605/OSF.IO/F2U78).

## 5 Results

The descriptive statistics of the GPS watches for distance covered measurements are shown in Table 1.

**Table 1:** Descriptive statistics.


	Total Distance Covered			
	Session-1- GPS Watch - 1	Session-1-GPS Watch - 2	Session-2- GPS Watch - 1	Session-2-GPS Watch - 2
Mean	1200.190	1199.952	1199.952	1200.048
Std. Deviation	0.814	0.218	0.218	0.498
95% CI Lower	1199.000	1199.000	1199.000	1199.000
95% CI Upper	1203.000	1200.000	1200.000	1202.000

### 5.1 Reliability of total distance measurement of GPS watches

Table 2 shows the inter-session reliability of the total distance traveled from the M 400 h. Session-1- GPS Watch – 1 and Session-1-GPS Watch – 2; (CV% = 0.05, SWC = 0.01, SEM = 0.09); Session-2- GPS Watch – 1 and Session-2-GPS Watch – 2; (CV% = 0.03, SWC = 0.01, SEM = 0.05); Session-1- GPS Watch – 1 and Session-1-GPS Watch – 1; (CV% = 0.05 SWC = 0.01 SEM = 0.09); Session-2- GPS Watch – 2 and Session-2-GPS Watch – 2; (CV% = 0.03, SWC = 0.01, SEM = 0.05).

**Table 2:** Between-session reliability of total distance covered, variables obtained from the GPS watch.


	Total Distance Covered			
Parameters		CV% ICC	SWC	SEM
Session-1- GPS Watch - 1	Session-1-GPS Watch - 2	0.05 0.95	0.01	0.09
Session-2-GPS Watch - 1	Session-2-GPS Watch - 2	0.03 0.94	0.01	0.05
Session-1- GPS Watch - 1	Session-2-GPS Watch - 1	0.05 0.96	0.01	0.09
Session-2-GPS Watch - 2	Session-2-GPS Watch - 2	0.03 0.95	0.01	0.05

**CV%:** Coefficient of variation; **ICC:** Intraclass correlation coefficient; **SWC:** Smallest Worthwhile Change; **SEM:** Standard error of measurement.

### 5.2 Validity of total distance measurement of GPS watches

**Fig. 3:**
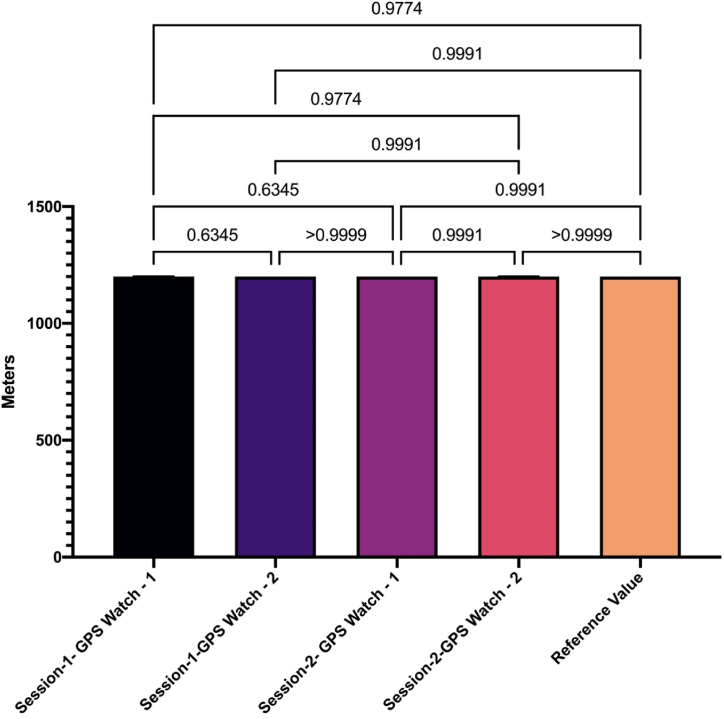
Reference value and intra-GPS watches differences.

**Fig. 4:**
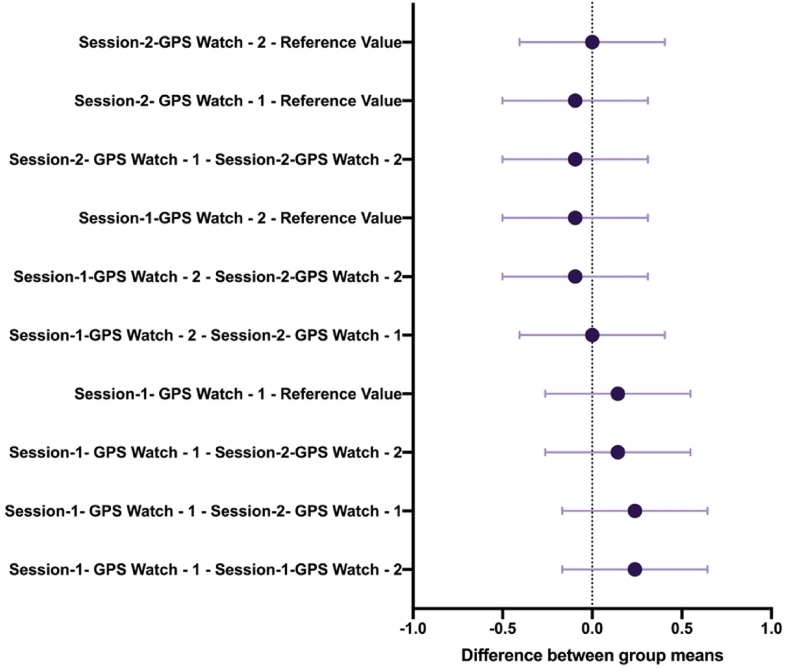
Difference between group means of reference value and intra-GPS watches.

Table 3 shows the difference between the reference value and the values measured by the watches (F = 1.086; p = 0.368; $\eta_{p}^{2}$ ≤0.042). Table 4 shows the individual differences between the reference value and the values measured by the watches. Reference values and Session-1- GPS Watch – 1; (SE = 0.142; p = 0.958); Reference values and Session-1-GPS Watch – 2; (SE = 0.142; p = 1.000); Reference values and Session-2- GPS Watch – 1; (SE = 0.142; p = 1.000); Reference values and Session-2-GPS Watch – 2; (SE = 0.142; p = 1.000). Fig. 3 shows the reference value and the time differences in GPS. Fig. 4 shows the difference between group means of reference value and intra-GPS watches.

**Table 3:** ANOVA - total distance covered.


Cases	Sum of Squares	df	Mean Square	F	*p*	η^2^
Group	0.914	4	0.229	1.086	0.368	0.042
Residuals	21.048	100	0.210

**Table 4:** Post hoc comparisons- group.


		Mean Difference	SE	t	*p*
Reference Value	Session-1- GPS Watch - 1	−0.238	0.142	−1.682	0.958
	Session-1-GPS Watch - 2	2.345e-15	0.142	1.657e-14	1.000
	Session-2- GPS Watch - 1	1.096e-15	0.142	7.744e-15	1.000
	Session-2-GPS Watch - 2	−0.095	0.142	−0.673	1.000

**Fig. 5:**
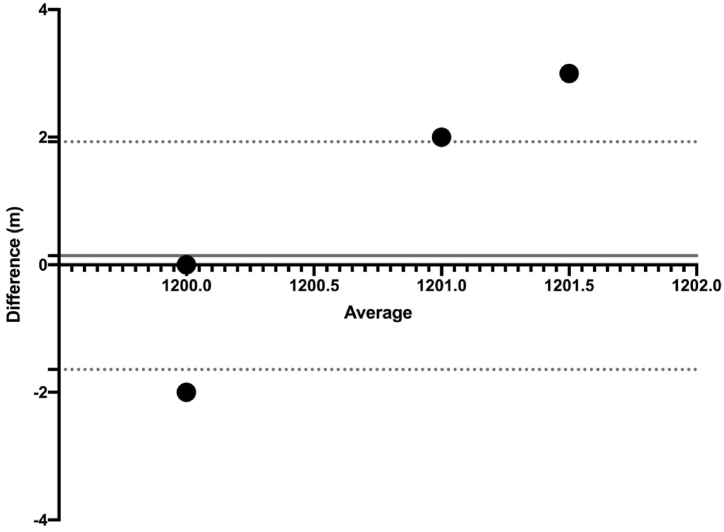
Bland - Altman plot of the reference distance and GPS watches distance.

The differences between the reference values and GPS watches distance, the upper limit, the lower limit and the mean bias values are shown in Fig. 5 with the Bland -Altman Plot. The biases of the measurements, the upper and lower limit of agreement are shown.

## 6 Discussion

This study aimed to analyze the validity and reliability of the Polar M400 watch for measuring distances covered in team sports. The main findings were that the Polar M400 watch (i) accurately measured the distances covered during the TSSC test; (ii) presented good inter-unit reliability levels during the TSSC test; and (iii) presented good inter-session reliability levels during the TSSC test. These results suggest that the Polar M400 watch is valid and reliable for measuring the distances covered in team sports. For simplicity, this discussion is divided into subsections concerning the validity and reliability of the Polar M400 watch for measuring distance, as well as the limitations of the study and its practical applications.

### 6.1 Validity of the polar M400 watch for measuring distance

The validity of the Polar M400 watch was examined against the gold standard (reference values) of the exact distance covered during the TSSC test [23,24]. The Polar M400 watch showed accurate values as no significant differences were revealed between the total distance measured by the watch and the real distance of the TSSC test. A recent study analyzed the accuracy of eight sport watches (three from Polar brand) to measure the distances covered on urban areas, on a forest and on a track and field, with fixed distances as reference [31]. That study [31], revealed that from the eight examined watches, the three Polar watches (V800, Vantage M and Vantage V) had the most accurate values, with the V800 model being the most accurate of all analyzed watches in all terrains. In the present study, standard error values of 0.1, and no significant systematic error (<-0.2 m) were revealed for both the Polar M400 watches in both sessions, when compared to the reference values of the TSSC test. These values are even lower than the findings of Gilgen-Ammann et al. [31], where a systematic error of 3.9 m was found for the V800 Polar watch when compared to the reference values.

Another study conducted on thirty-six amateur endurance athletes tested the validity of the Polar Ignite and the Garmin Forerunner 945 watches for measuring distance [32]. That study revealed significant deviations for the Polar Ignite watch (min: −50 m; and max: 150 m) and for the Garmin Forerunner 945 watch (min: −30 m; and max: 90 m) for a 1.6 km interval run in an outdoor track and field [32]. Despite such deviations, the authors stated that given the relatively low values of the mean absolute error (MAE) and mean absolute percentage error (MAPE) for both watches, “the deviations were negligible for practical use” [32]. These findings are in contrast with the results of the present study and those reported from the study of Gilgen-Ammann et al. [31]. Interestingly, both Gilgen-Ammann et al. [31], and Budig et al. [32] studies, revealed that all tested watches had an overestimation (higher error) of the distance covered in both urban and forest contexts when compared to a outdoor track and field/stadium. Both authors suggested that this could be explained by a lack of satellites signal when running between high buildings and/or in courses with high and dense vegetation [31,32].

Previous studies tested the accuracy of other bracelets and sports watches and different accuracy levels were reported depending on the brand and model used [[33], [34], [35]]. However, none of them were tested on team sports contexts, and to the best of our knowledge, no study tested the validity of the Polar M400 watch for measuring distance. Our results confirmed that the Polar M400 watch is a valid tool to measure distances in open-field areas.

### 6.2 Reliability of the polar M400 watch for measuring distance

Considering the inter-session reliability of the Polar M400 watch, the very low and narrow range values of both %CV (0.03–0.05%) and SEM (0.05–0.09) found in the present study, confirmed its ability to consistently measure total distance by team sport players. Indeed, %CV values below 5 % has been categorized as good reliability when considering the typical errors of GPS devices used in team sports [11]. A recent systematic review examined the validity and reliability of discontinued and current commercially available watches for measuring step count, HR, and energy expenditure [36]. In that systematic review [36], the authors stated that fewer studies examining the reliability of sport watches exist in comparison to validity studies. Although the authors of that study revealed very strong inter-device reliability for all selected devices, no study examined intra-device reliability [36]. Interestingly, a very recent study revealed that the Polar V800 watch presented coefficients of variations below 5 % for measuring vertical jump performance [37]. Still, to the best of our knowledge, no study tested the inter-unit and inter-session reliability of a sport watch for measuring distance, including the Polar M400. These facts make comparisons with other studies difficult.

Despite that, and given the extensive use of wearable GPS devices in team sports, which includes gyroscopes, magnetometers and triaxial accelerometers in the same device to measure motion [26], a brief discussion can be opened regarding their reliability levels. Indeed, some of those type of GPS were previously examined for their reliability levels for measuring the distances covered by team sport players, among other time-motion variables [[38], [39]]. In fact, in the study of ▣Gray et al. [39], intra- and inter-device coefficient of variation (2.66▣% and 2.88 %, respectively) were less than 5 % for all analyzed movements (walk, jog, run and sprint) in both linear and non-linear delineated courses for testing. However, the authors found that more variability is seen during non-linear courses and at higher intensity movements such as sprinting [39].

It was previously stated that 1–15 Hz devices allow reliable quantification of total distance covered [38]. However, total distances covered does not consider the different speed thresholds of motion. Similar to the findings of ▣Gray et al. [39], Hoppe et al. [38], confirmed that devices with lower sample rates present lower reliability levels when measuring higher speed movements. The TSSC test used in the present study also included acceleration, deceleration, sprinting, walking, jogging, and changes of direction [23,24]. However, only the total distance covered was analyzed, as examining the different speed thresholds (0–36 km/h) available on the Polar M400 watch was out of this study aims. Nevertheless, the Polar M400 watch revealed to be a reliable option for measuring total distances covered by team sport players.

### 6.3 Study limitations

The present study presented some limitations. The main limitation was regarding the sample size as only twenty-one team sport athletes were included in the analysis. The small sample size can potentially impact the generalizations of our findings for all team sports contexts. Although the Polar M400 is widely used by coaches and athletes from lower-budget contexts, to be able to monitor training activities, it is currently discontinued. Future studies should test the validity and reliability of currently commercially available sport watches to monitor training activities. Moreover, we did not analyze the different speed thresholds allowed by the Polar M400. Future studies that can test available sport watches, should examine the different speed intensities recorded and include athletes from different team games.

### 6.4 Practical applications

Despite all the above-mentioned study limitations, the present study has some practical applications to the team sports community. Indeed, the good levels of accuracy and the strong precision levels reported in this study for the measurement of total distances covered by the Polar M400 watch can ensure to their actual users that they can securely monitor their training activity. In many team sports contexts, the use of expensive equipment such as wearable GPS with sample rates of 10 or 18 Hz, are not an option. Given that, the use of wrist sports watches such as the Polar M400, are a less expensive option to accurately and precisely measure the total distances covered by team sport players.

## 7 Conclusions

The Polar M400 sport watch was shown to be a valid and reliable device for measuring the total distances covered by team sport athletes in an outdoor field. The absence of significant differences between the and the Polar M400 sport watch and the reference value, determined that the Polar M400 is a useful and inexpensive device for the assessment of total distances covered, at least during a team sport simulation test. Future studies are needed on different devices and in different sports.

## Declarations

**Ethics approval and consent to participate:** The study was conducted according to the guidelines of the Declaration of Helsinki and approved by the Afyon Kocatepe University Review Board Approval Number: 2021-2). All experimental protocols were approved by Afyon Kocatepe University Ethics Committee. Informed consent to participate was obtained from all participants.

## 8 Consent for publication

Not Applicable.

## Funding

This work had no funding.

## Data availability statement

Data associated with present study been deposited into a publicly available repository. The data presented in this study are available on website: https://osf.io/f2u78/with Identifier: DOI: 10.17605/OSF.IO/F2U78.

## CRediT authorship contribution statement

**Piotr Makar:** Writing – review & editing, Writing – original draft, Methodology, Conceptualization. **Adam Kawczyński:** Writing – review & editing, Methodology, Investigation, Formal analysis. **Rui Miguel Silva:** Investigation, Data curation. **Mehmet Yildiz:** Methodology, Formal analysis, Data curation. **Ana Filipa Silva:** Supervision, Methodology. **Zeki Akyildiz:** Writing – review & editing, Writing – original draft, Supervision, Project administration, Conceptualization.

## Declaration of competing interest

The authors declare that they have no known competing financial interests or personal relationships that could have appeared to influence the work reported in this paper.
